# Interleukin-21 rs2055979 and Interleukin-21 receptor rs3093390 genetic variants and hepatitis C virus chronic infection 

**Published:** 2017

**Authors:** Shahrzad Shoraka, Seyed Reza Mohebbi, Seyed Masoud Hosseini, Armin Hosseini Razavi, Amir Ghaemi, Shabnam Kazemian, Mohammad Rostami-Nejad

**Affiliations:** 1 *Basic and Molecular Epidemiology of Gastrointestinal Disorders Research Center, Research Institute for Gastroenterology and Liver Diseases, Shahid Beheshti University of Medical Sciences, Tehran, Iran*; 2 *Department of Microbiology and Microbial Biotechnology, Faculty of Life Sciences and Biotechnology, Shahid Beheshti University, Tehran, Iran*; 3 *Gastroenterology and Liver Diseases Research Center, Research Institute for Gastroenterology and Liver Diseases, Shahid Beheshti University of Medical Sciences, Tehran, Iran *; 4 *Department of Virology, Pasteur Institute of Iran, Tehran, Iran*; 5 *Foodborne and Waterborne Diseases Research Center, Research Institute for Gastroenterology and Liver Diseases, Shahid Beheshti University of Medical Sciences, Tehran, Iran*

**Keywords:** Hepatitis C virus, Interleukin-21, Polymorphism, Single Nucleotide

## Abstract

**Aim:**

The aim of this case-control study was to investigate association of G/T IL-21 (rs2055979) and C/T IL-21R (rs3093390) gene polymorphisms with chronic hepatitis C virus in Iranian Patients.

**Background:**

Interleukin 21 (IL-21) has a significant function in the regulation of cellular immune responses. Its exclusive receptor, IL-21R, expressed on the surface of T, B and NK cells and is important for the proliferation and differentiation of these immune cells. Hence, it was suggested to be involved in response to viral infections.

**Methods:**

This study follows a case-control study design and blood samples were collected from 290 patients with chronic HCV and 290 controls for both genes. Genomic DNA was extracted and then for each position, SNP was genotyped by the dedicated PCR and restriction fragment length polymorphism (RFLP) method. The results were analyzed by SPSS software using logistic regression and Chi-square tests.

**Results:**

Genotype frequencies of GG, GT and TT in IL21 gene (rs3093390) were found to be 27.6%, 48.3%, 24.1% and 25.2%, 55,5%, 19,3% respectively in HCV infected patients and control group. For IL21R gene (rs2055979) genotype frequencies of CC, CT and TT were 63.8%, 31.4%, 4.8% and 61.4%, 29.7%, 9.0% respectively in HCV infected patients and control group. P values for genotype and allele frequencies were p=0.188, p=0.769 for IL21 gene, and p=0.144, p=0.179 for IL21R gene respectively.

**Conclusion:**

As a result, there is no evidence for an association between IL-21 (rs2055979) and IL-21R (rs3093390) gene polymorphisms with chronic hepatitis C virus in Iranian Patients.

## Introduction

 Hepatitis C virus infection has become global health concern. More than 184 million people have been infected with hepatitis C virus (HCV) in the world (1, 2). Hepatitis C can contribute to persistent infection in a chronic disease in most of the patients and also can progress to fibrosis, cirrhosis and hepatocellular carcinoma (HCC) (3). HCV is also one of the main causes of liver transplantation around the world (4). Recent studies have found that viral, host, and environmental factors may involve in susceptibility to HCV chronic infection (5).

The vigor of the immune response plays an important role in the outcome of viral infections (6). Cytokines as key components of immune system are intercellular mediators involved in viral control and damages being induced by infection (7). Interleukin-21 (IL-21) is the most recently discovered member of type-I cytokine superfamily that has regulatory effects on the immune system. Interleukin 21, mainly produced by a range of differentiated CD4+ T-cell subsets (8). IL-21 specifically sustains CD8+ T cell effector activity and prepares a mechanism of CD4+ T cell helper during chronic viral infections (9). It also has a significant influence on the regulation of B-cell functions. It promotes the differentiation of antigen-stimulated B cells into memory and antibody-secreting plasma cells, affects IgE production, and induces Ig switching to IgG1 and IgG3 production (10). Also, several in vivo studies in animal models have demonstrated that IL-21 is vital for controlling chronic viral infections (11, 12). On the other hand, its private receptor, IL-21R, shares the common γ-chain with IL-2R, IL-4R, IL-7R, IL-9R, and IL-15R. Engagement of IL-21 to its cognate receptor activates Jak1, Jak3 and subsequently STATs notably STAT1, STAT5a, STAT5b and predominantly STATE. Besides, IL-21 signals through the canonical PI3K and MAPK pathways(13). As multiple immune cell types express the IL21R, this cytokine exerts pleiotropic modulatory functions in both innate and adaptive cells including NK cells, B cells, CD4 and CD8 T cells (13, 14). 

Among the host factors, single nucleotide polymorphisms (SNPs) in immune genes such as interleukins can play significant roles in regulation the host immune response to the viral infections (15). In addition, some studies demonstrated that single nucleotide polymorphisms in cytokines and their receptor genes may contribute to the susceptibility of disorders and diseases (11, 16-22). Recently, many studies supported that variant SNPs located within the IL-21 gene have genetic association with susceptibility to different diseases particularly autoimmune diseases (18, 23, 24). 

Herein, in the present study, association of intronic polymorphisms in IL-21 gene (rs2055979) and its dedicated receptor (rs3093390) with chronic HCV infection was distinguished. 

## Methods

**Subjects**

In this Case-control study, for each gene, we analyzed a total of 580 subjects, including 290 chronic HCV patients who admitted in Gastroenterology and Liver Diseases Research Center, Shahid Beheshti University of Medical Sciences, and also 290 healthy individuals as a control group.

**Serological Assays**

Control group were healthy individuals without any liver diseases along with negative results for anti–HCV antibody tested by ELISA and then the presence or absence of viral particles in serum was determined by reverse transcription-PCR (RT-PCR). Selection criteria for patients group were positive results for anti–HCV antibody ELISA. RT-PCR and HCV RNA PCR were tested for anti HCV antibody positive cases.

**Table1:** The PCR-RFLP products size, restriction site of enzymes and genotypes for each SNP

Gene	SNP	PCR product size (bp)	Restriction enzyme	Restriction site	RFLP fragments size (bp)	Genotype
IL-21	rs2055979	490	NIa III	5’…CATG…3’ 3’…GTAC…5’	490	TT
					490, 328, 162	GT
					328, 162	GG
IL-21R	rs3093390	182	MvaI	5’…CCWGG…3’ 3’…GGWCC…5’	182	TT
					182, 118, 65	CT
					118, 65	CC

**Genomic DNA extraction and genotyping**

Total Genomic DNA was extracted from EDTA-anticoagulated peripheral blood samples of both patients and control group by the standard salting-out method(25). For each studied gene, polymorphic region was amplified by the polymerase chain reaction (PCR) in a total volume of 25 μL, using PCR reagents and specific primers. The annealing temperature (Tm) for the PCR was 62°C for IL21 and 61.2°C for IL21R gene. Then the PCR products were analyzed by electrophoresis on 1.5% agarose (Roche, Germany) gel to confident that gene fragment containing the considered SNP was amplified. Then 10 microliters of the PCR products were digested with the NIaIII (BioLabs) restriction enzyme for IL21 gene and the MvaI (Fermentas) for IL21R gene. After 16 hours incubation in a 37° centigrade water bath, the RFLP products were analyzed by 3% agarose (Roche, Germany) gel. Additionally, to confirm the PCR-RFLP assay, in each position, about 10% of total samples with different kinds of genotypes were selected and then their PCR products were sequenced. The PCR-RFLP products size, restriction site of enzymes and genotypes, for each SNP are shown in Table 1.

**Statistical analyses**

Genotype and allele frequencies of IL-21 and IL-21R were compared between HCV infected cases and controls using the Chi-square (χ^2^) test. To adjust for confounding factors including age and gender, logistic regression analysis was used. To estimate the association between individual polymorphisms and chronic HCV, we were used odds ratios (OR) and 95% Confidence Intervals (95% CI). P-values of less than 0.05 were considered statistically significant. All statistical analyses were performed using the SPSS software (version 22; SPSS, Chicago, IL, USA).

## Results

In this study, the case group consisted of 290 chronic HCV patients and the control group consisted of 290 healthy individuals. The number of males and females participated in this study is shown in table 2 based on case and control groups for both genes. Mean age for all individuals are calculated and the results are shown in table 3. 

**Table 2:** The number of males and females participated in this study

Gene	Status	Gender		Total	
		Male	Female		
IL21	Control	137	153	290	580
	HCV+	222	68	290	
IL21R	Control	135	155	290	580
	HCV+	224	66	290	

About 10% of total samples with different kinds of genotypes were selected and then their PCR products were rechecked by the sequencing method and were concordant with the original RFLP results. Result of sequencing for heterozygous genotypes are shown in Figure 3.

**Table 3:** Mean age for all individuals in this study

Groups		Mean
Il21	HCV+	43.70
	Control	40.32
Il21R	HCV+	43.70
	Control	40.32

**Figure 1.**
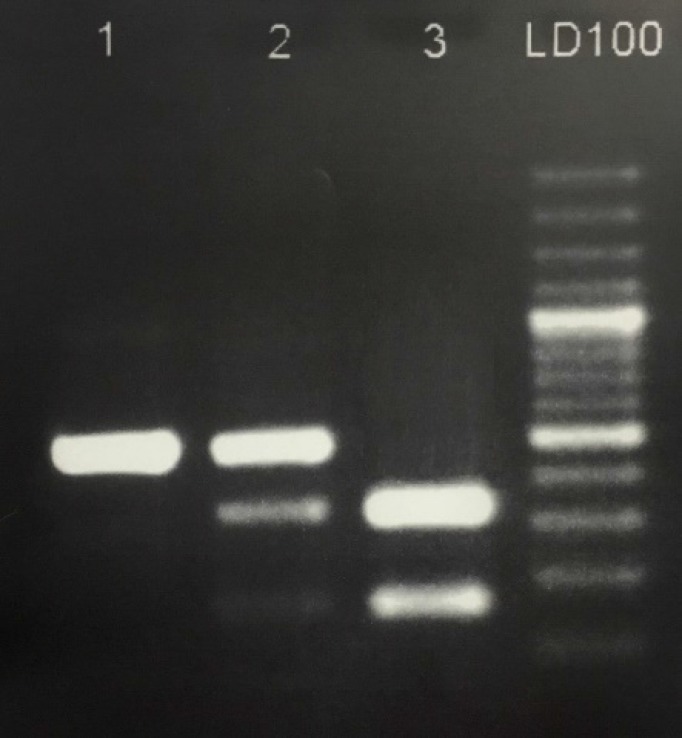
Genotype of polymorphism (rs2055979) in IL-21 gene revealed by PCR-RFLP on 3% agarose gel. Lane 1 is major homozygous TT (490bp), lanes 2 is heterozygous GT (490, 328, 162bp) and lane 3 is minor homozygous GG (328, 162bp

**Table 4:** Genotype and allele frequency of IL-21 and IL-21R polymorphisms among patients and control groups

Genotype	Controls no(%)	Chronic HCV no(%)	P value
rs2055979	290	290	0.188
GG	80(27.6%)	73(25.2%)	
GT	140(48.3%)	161(55.5%)	
TT	70(24.1%)	56(19.3%)	
rs3093390	290	290	0.144
CC	178(61.4%)	185(63.8%)	
CT	86(29.7%)	91(31.4%)	
TT	26(9.0%)	14(4.8%)	
rs2055979	580	580	0.769
G	307(52.9%)	302(52.1%)	
T	273(47.1%)	278(47.9%)	
rs3093390	580	580	0.179
C	442(76.2%)	461(79.5%)	
T	138(23.8%)	119(20.5%)	

**Figure 2:**
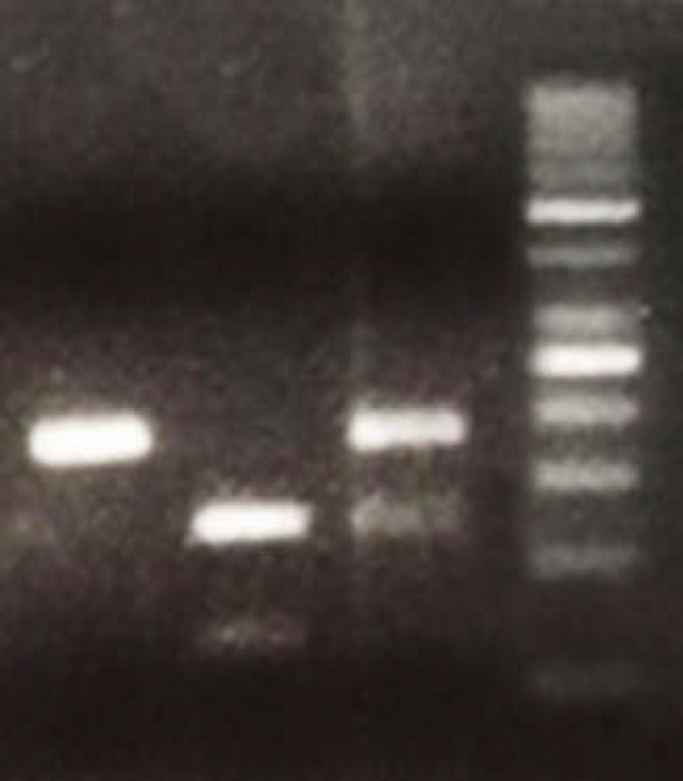
Genotype of polymorphism (rs3093390) in IL-21R gene revealed by PCR-RFLP on 3% agarose gel. Lane 1 is homozygous TT (182bp), lanes 2 is homozygous CC (118, 65bp) and lane 3 is heterozygous CT (182, 118, 65bp

**Figure 3:**
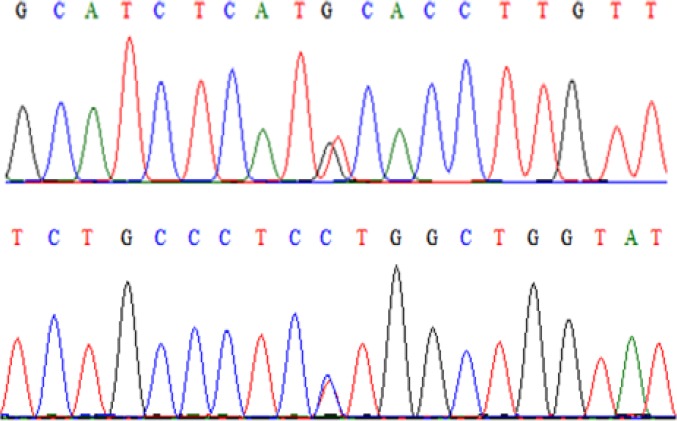
Result of sequencing. DNA sequence of Heterozygous A) GT for IL21 gene B) CT for IL21R gene are shown by spear

The genotype and allelic frequencies of IL-21 and IL-21 receptor genes variation of the patient and control groups are presented in Table 4. The chi-square test was used to analyze the association between the genotypes and hepatitis status, the result is also shown in Table 4.

According to result of the Chi-square test, there were no statistically significance in the distribution of genotypes and allele frequencies between the patients and control groups for IL21 (rs2055979) and IL21R (rs3093390) gene (P >0.05). P values for genotype and allele frequencies were p=0.188, p=0.769 for IL21 gene, and p=0.144, p=0.179 for IL21R gene respectively.

The mean age of the HCV patients was greater than individuals in healthy controls, also the percentage of men was higher in the HCV group than in the healthy control group; therefore, we used the logistic regression method to omit these confounding variables. After adjustment for covariates including age and gender, in both gene, there is no significant difference between the cases and controls. Results are shown in table 5.

**Table 5:** Adjustment for covariates including age and gender using logistic regression

Genotype	Regression (Adjusted)		
	OR	95%CI	P value
rs2055979			
GG	1.00 (reference)		
GT	1.019	0.674-1.540	0.929
TT	0.778	0.471-1.287	0.328
rs3093390			
CC	1.00 (reference)		
CT	1.050	0.716-1.540	0.803
TT	0.634	0.310-1.297	0.212
Allele	Regression (Adjusted)		
	OR	95% CI	P value
rs2055979			
G	1.00 (reference)		
T	0.891	0.663-1.198	0.466
rs3093390			
C	1.00 (reference)		
T	0.911	0.713-1.163	0.455

## Discussion

In our study, we genotyped two SNPs and evaluated the association of these SNPs with chronic HCV infection. We found no association between IL21 (rs2055979) and IL21R (rs3093390) and susceptibility to chronic HCV infection. To our knowledge, no more extra studies were found about the association of IL21 (rs2055979) and IL21R (rs3093390) with chronic HCV infection in Iranian population.

Single nucleotide polymorphisms (SNPs) are the most frequent kinds of genetic variations (26). Many studies supported that SNPs may increase or decrease susceptibility to various diseases(26, 27). Cytokines play critical roles in immune responses. On the other hand, cytokine coding genes are polymorphic and some of these polymorphisms may affect the expression of cytokines and also can play roles in the severity and progression of immune-mediated and chronic inflammatory diseases (21). Furthermore, the essential roles of the interleukin receptor genes in function of their exclusive interleukins are not hidden (28, 29). 

IL-21 is a pleiotropic cytokine has been shown to play a significant role in the CD8 T cell response during acute and chronic viral infections. Although the role of IL-21 signaling in the CD4 T cell response to viral infection remains imperfectly defined (30). Recently, there is growing evidence that IL-21 leads to the pathogenesis of chronic inflammatory and autoimmune diseases due to its biological functions (31, 32). In addition, the important role for IL-21 as a major helper cytokine during acute HCV infection are proved (11, 33). Also lower serum IL-21 levels in chronic HCV patients are detected(34). IL-21 binds to the IL-21 receptor and then can activate some signaling pathways like; Janus kinase (JAK)1, JAK3, STAT1, and STAT3 (14). Recently, discoveries of loss-of function (LOF) mutations in IL-21R in humans have manifested unexpected roles for IL-21 in immune regulation (28).

Various single nucleotide polymorphisms within IL-21 and also IL-21R gene are found. Also the association between these polymorphisms and several diseases have been investigated previously. Many studies have investigated associations between genetic variations in these genes and viral infection, but results of these studies have been inconsistent (28, 29). R. nohra et al. have proved that variants of IL21R demonstrated association with MS and several of the SNPs in the associated region of IL21R can potentially affect the transcription factor-binding sites (35). 

17 SNPs are genotyped by Ryan Webb et al. in 2009; these SNPs are located within the IL21R gene. They found no evidence of a synergistic effect of the presence of the risk alleles in rs907715 and rs3093301 (P = 0.58), therefore there is no genetic epistasis between the IL21 and IL21R loci in lupus susceptibility (20). In 2013 Jian Zhang et al. identified and confirmed a higher prevalence of A alleles of IL-21 rs2221903 SNP in Graves’ disease patients and also for the IL-21R gene, compared with controls, the genotype frequencies of rs3093301 and rs2285452 were significantly different in Hashimoto’s thyroiditis (HT) patients. In addition, they revealed that may higher risk of HT in individuals with the SNPs of the common IL-21 and IL-21R (18). Na Li et al. demonstrated that IL21 rs2221903 by interacting with IL21R rs3093301, was associated with serum IL-21 and IgE levels in HBV chronic infected patients. It is declared that the susceptibility to and/or persistence of HBV infection potentially may be related to IL21 rs2221903 and IL21R rs3093301 polymorphisms through modifying the IL-21 and IgE production (36). Recently, Ching-Sheng Hsu et al. demonstrated a clinically effective role of IL-21R genotype in predicting IFN-based therapeutic response in Asian chronic hepatitis C patients with unfavorable IL28B genotype. In other words, among patients with IL28B rs8099917 non-TT genotypes, patients with IL-21R rs3093390 CC genotype had a higher sustained virologic response (SVR) rate than those with non-CC genotypes (CC versus non-CC 14/24 versus 0/4; P=0.031) (37). Amal Ahmed Mohamed et. al in 2016 distinguished that Egyptian patients with IL-21R rs3093390 CC genotype had a higher sustained virological response (SVR) to interferon treatment than those with non-CC genotypes(38). 

The association between genetic variants, defective serum cytokines, improper immune response and susceptibility to various diseases is a topic which has been investigated intensively for hepatitis C and B infection. Recent studies proved that IL-21R polymorphisms are the strongest predictors of sustained virological response to HCV. In addition, IL-21 and IL-21R polymorphisms can be affected on susceptibility for HCV infection in different populations. However, this matter was influenced by viral load and HCV genotype. 

Up to now, our data and others do not support that IL-21(rs2055979) and IL21R (rs3093390) genetic variants have an association with chronic HCV infection. Since only two SNPs were genotyped, and a small patient sample size was involved in the present study, to confirm this conclusion, further studies with more SNPs and larger patient sample sizes are required.

## Conflict of interests

The authors declare that they have no conflict of interest.
